# Recurrent hematuria involving urinary system with Klippel-Trenaunay syndrome: A case report

**DOI:** 10.1097/MD.0000000000036923

**Published:** 2024-02-16

**Authors:** Feng Lin, Kewei Yang, Jiadong Xu, Gang Wang, Lixia Yang, Jinrong Huang, Dan Li

**Affiliations:** a Department of Urology, Affiliated Hospital of Shaoxing University, Shaoxing, Zhejiang, China.

**Keywords:** hematuria, Klippel-Trenaunay syndrome, urinary system, varicose veins of lower extremities, venous malformations

## Abstract

**Rationale:**

Klippel-Trenaunay syndrome (KTS) is a rare congenital venous malformation, it had been found to be caused by mutations of the phosphatidylinositol 4, 5-diphosphate 3-kinase catalytic subunit alpha (PIK3CA) gene. Currently KTS is defined as a triad of skin wine pigmented spots, varicose veins and malformations of the lower extremities, and hypertrophy of bone and soft tissue, involving urinary system up to 6% to 30%. When the urinary system is involved, KTS is often presented as painless massive gross hematuria.

**Patient concerns:**

This article describes a woman who was hospitalized with painless massive gross hematuria. Physical examination revealed significant hypertrophy of the right lower limb with varicose veins, port-wine stains in the skin, and right perineal hemangiomatous changes with swelling. The patient was admitted to hospital 4 times for repeated hematuria and infection.

**Diagnoses:**

By physical examination, CT urography, ureteroscopy and cystoscopy, the patient was diagnosed to have Klippel-Trenaunay syndrome, involving the urinary system.

**Interventions:**

The patient hematuria improved after multiple indwelling D-J tubes and anti-inflammatory treatment.

**Outcomes:**

The final symptoms of hematuria improved significantly, follow-up so far has not recurred.

**Lessons:**

This case presents the possibility of painless gross hematuria with KTS. Most of patients can be improved by conservative treatment. Cystoscopic laser therapy is the preferred treatment for poor bleeding control. Cystectomy and nephrectomy should be considered when life-threatening.

## 1. Introduction

In 1900, Klippel and Trenaunay in France first reported the disease characterized by soft tissue and bone hypertrophy, varicose veins and malformations of lower extremities, and skin wine pigmented spots, namely Klippel-Trenaunay syndrome (KTS).^[1]^ KTS is mainly a mixed mesodermal abnormality, presenting as early as childhood and adolescence.^[2]^ The mean age of diagnosis is 4.9 years, and the mean age of urinary complications and involvement is 7.6 years.^[3]^ About 75% of patients develop symptoms before the age of 10 years.^[4]^ The genitourinary system has various manifestations, affecting the kidney, bladder, scrotum and perineum.^[5]^ The degree of hematuria varies and even causes hemorrhagic shock in severe cases.^[4]^ The main endoscopic manifestations are hemangiomatous lesions of kidney and bladder with extensive hemorrhagic sites, and may also be complicated with lymphomatoid lesions. Most can be relieved with conservative treatment. If bleeding persists, endoscopic laser therapy is the best choice for bladder bleeding lesions. In cases of uncontrolled bleeding, partial and subtotal cystectomy and nephrectomy may be considered.^[6]^

Reported here is a middle-aged woman with painless gross hematuria. The physical examination was consistent with the KTS triad. Combined with endoscopic findings, we considered that the patient had KTS and involved the urinary system. After multiple placements of double J tubes and anti-inflammatory, hematuria was controlled, and there has been no recurrence so far.

## 2. Case presentation

**Figure 1.**
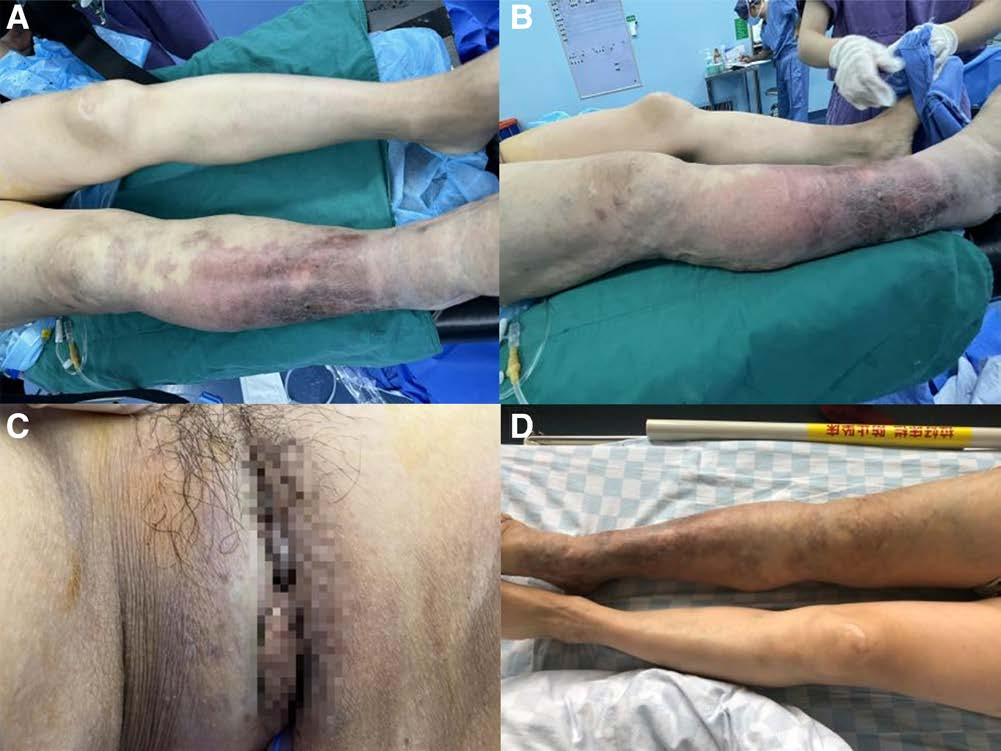
(A) Port-wine stains of skin. (B) Significant hypertrophy of the right lower extremity. (C) hemangiomatous change of the right perineum with swelling. (D) Varicose veins in the right lower limb.

A 55-year-old female patient was hospitalized on June 30, 2021 with “painless gross hematuria for 1 week.” The patient had a history of congenital varicose veins and venous valvular insufficiency in the right lower limb, as well as surgical history of varicose vein dissection in the right lower limb. Previous venography showed superficial varicose veins. The patient self-reported that the skin pigment of the right lower limb was changed at birth, varicose veins were abnormal at age 17, and gross hematuria appeared at age 30, while no corresponding disease was found in his immediate family, including his parents and children. Physical examination revealed wine-red patchy changes in the patient right lower limb, obvious hypertrophy of the right lower limb with varicose veins, and hemangiomatous changes with swelling in the right perineum, as shown in Figure 1.

**Figure 2.**
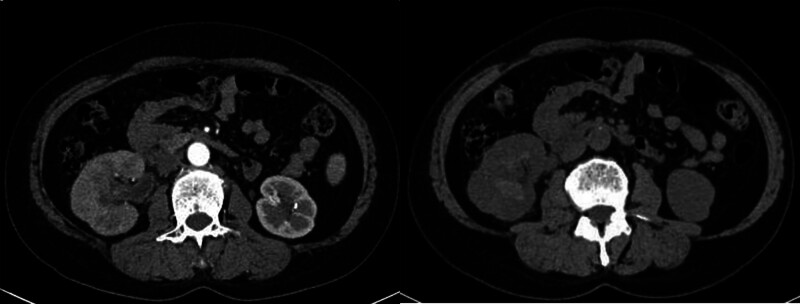
The right kidney was weakened, the renal cortex was swollen, and the upper wall of the right ureter was thickened.

**Figure 3.**
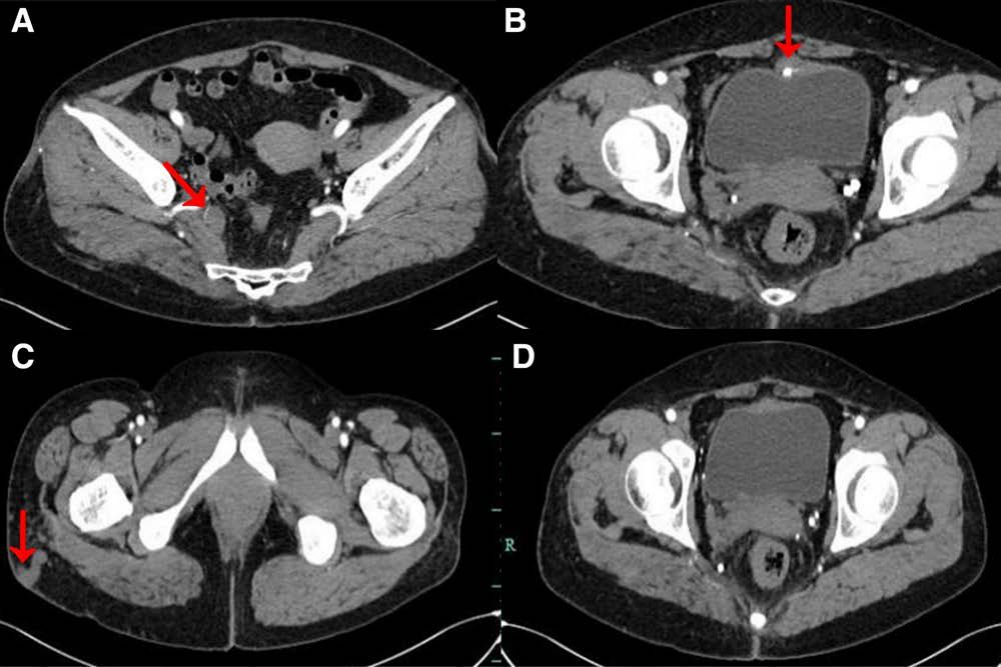
(A) Obvious thickening of the right internal iliac vein and its branches; (B) The top of the bladder is suspected to have thickened veins (C) Obvious thickening of the right hip vein and thickening of soft tissue; (D) large number of round calcification points in pelvic cavity and lower abdomen (phlebolithiasis).

**Figure 4.**
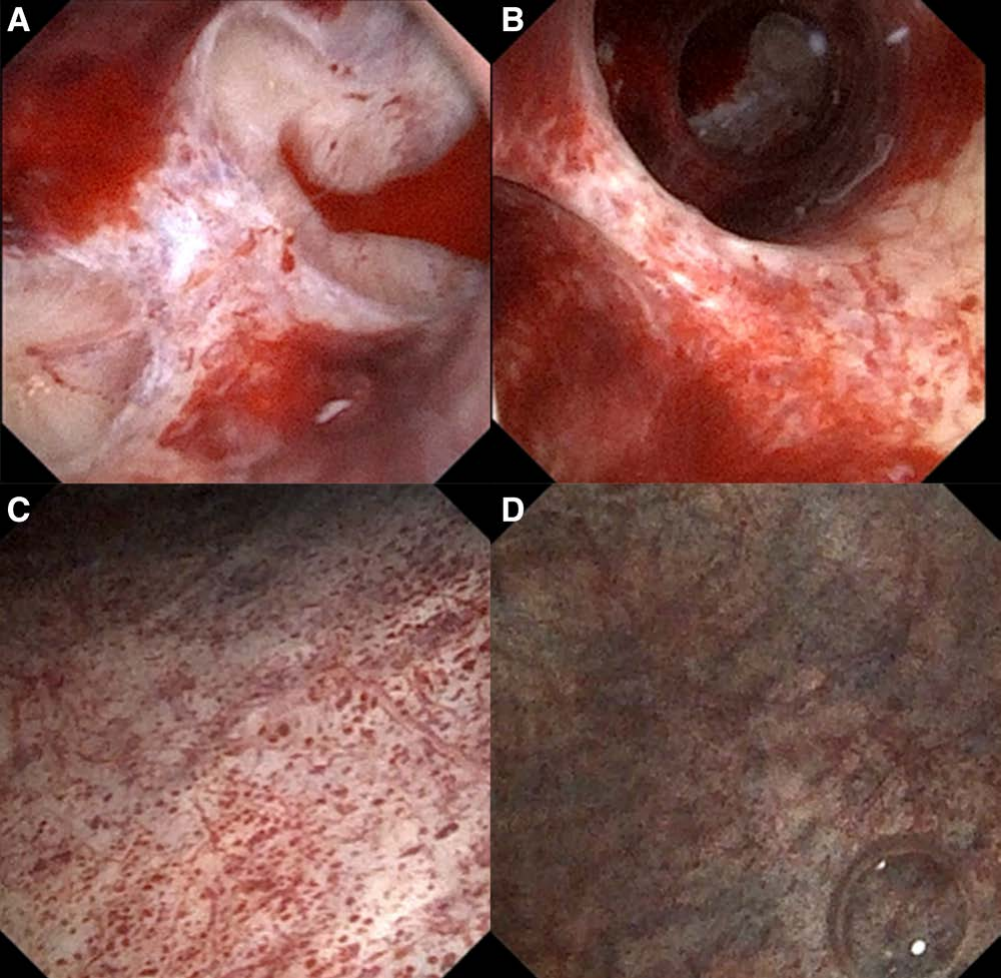
(A) Red blood clots in calyces. (B) Diffuse multiple bleeding foci in calyces. (C) Extensive bleeding spots and small hemangiomas in the bladder. (D) The internal vesical veins are tortuous.

The CT urography showed decreased strengthening of the right kidney, swelling of the renal cortex, thickening of the wall of the upper part of the right ureter, and no obvious occupying of the bladder, as shown in Figure 2. Multiple varicose venous malformations were found, mainly manifested as: The right internal iliac vein and its branch were thickened obviously; The right hip vein has increased significantly; A large number of round calcification spots (phlebiolith) in the pelvic and lower abdomen are shown in Figure 3. Ureteroscopy and D-J tube implantation were performed on July 12, 2021. During the operation, ureteroscopy showed diffuse multiple hemorrhagic points of renal pelvis and calyx with red clots. The bleeding points in the bladder were mainly distributed on the right side, and the left part was normal. Varicose veins were visible in the mucosa, and extensive hemorrhagic points and small hemangioma protruding from the bladder mucosa, as shown in Figure 4. This hematuria is unusual and seems to have some connection with varicose veins.

In August of the same year, she was admitted to the hospital again due to aggravation of hematuria. After CT urography review, there was no obvious swelling of the right kidney, which returned to normal. No significant changes were observed under endoscopy. The D-J tube was replaced and the hematuria improved. In July 2022, she was admitted to the hospital due to fever with gross hematuria, flet chills with the highest temperature of 40.1°C. Her right lower limb was swollen with redness and elevated skin temperature. Blood culture was positive for *Escherichia coli*. Acute lymphangitis of the right lower limb was considered, and the D-J tube was re-indwelled after anti-infection improvement of imipenem combined with vancomycin. In October 2022, infection occurred again in the right lower limb, hematuria improved after anti-inflammatory, and no indwelling D-J tube was placed.

According to the patient medical history, this hematuria was repeated, which was caused by internal bleeding of bladder and renal calyces, and since blood clots and bleeding spots were mainly distributed in the kidney, we believed that the bleeding was mainly in the kidney. The occurrence of hematuria is often related to the infection of the right lower limb vein, which is also consistent with the triad of KTS. Finally, we consider that the hematuria of the patient is caused by the accumulation of KTS in the urinary system and perineum.

## 3. Discussion

In 1900, Klippel and Trenaunay in France first reported the disease characterized by soft tissue and bone hypertrophy, varicose veins and malformations of lower extremities, and skin wine-pigmented spots, namely KTS syndrome.^[3]^ In 1907, Parkes-Weber independently presented several cases similar to those described by Klippel and Trenaunay. Parkes-Weber subsequently reported a case involving an arteriovenous fistula in the affected limb in 1918. Parkes-Weber syndrome can be distinguished from Klippel-Trenaunay syndrome due to obvious arteriovenous fistula and high-flow hemodynamic changes.^[7]^ Many KTS patients are born with “red wine” stained patchy changes in the upper or lower extremities, which is a skin capillary malformation. Hemorrhagic papules or pustules, varicose veins, and lymphatic malformations may also occur. There is usually serious venous blood flow of lower limbs through multidetector CT, MRI, ultrasound and other imageological examination, often can find abnormal thick lateral femoral drainage vein (permanent embryonic vein) and deep vein tumor, absence, dysplasia and venous insufficiency.^[8]^ KTS can invade various parts of the body and may be accompanied by vascular malformations in other organs, such as the spinal cord, abdominal organs, pelvic cavity, esophagus, intestine, vagina, perineum, bladder.^[5]^ Patel N retrospectively analyzed 58 patients with KTS, among whom 10 patients had genitourinary system involvement.^[3]^ Cystoscopy mainly presents with hemangiomatous lesions of the bladder with extensive hemorrhagic sites, but may also be complicated with lymphomatoid lesions.

KTS had been found to be caused by mutations in the somatic function acquisition activation of the phosphatidylinositol 4, 5-diphosphate 3-kinase catalytic subunit alpha (PIK3CA) gene. The PIK3CA gene appears to be involved in the disease process. The PIK3CA gene expresses an overactive phosphatidyl inositase, leading to “abnormal growth of bones, soft tissues, and blood vessels.”^[9]^ In 1975, Klein and Kaplan reported bladder hemangioma in children, the first confirmed case of Klippel-Trenaunay syndrome associated with urogenital hemangioma.^[10]^ In 1988, Campistol JM reported on a 19-year-old woman with KTS who developed severe unilateral upper urinary hematuria. Renal angiography revealed hemangioma of the left kidney and a left nephrectomy was performed.^[11]^ In 1994, Fligelstone in the UK reported a case of a 29-year-old male with KTS who eventually underwent right nephrectomy due to upper urinary tract active bleeding.^[12]^ In 2006, Rubenwolf in Germany reported on a 9-year-old boy with KTS who was admitted to hospital with life-threatening recurrent hematuria and underwent a subtotal cystectomy and enterocystoplasty using the ileocecal segment.^[5]^ In 2010, Beijing Tonge Hospital reported on a 20-year-old female patient with KTS who suffered hypovolemic shock due to bladder hemorrhage, HB decreased to 23 g/l, and underwent distal embolization of the right internal iliac artery. After surgery, she still had persistent hematuria, which was finally relieved by endoscopic laser treatment.^[6]^

Bladder vascular malformations caused by KTS often involve both the rectum or pelvic organs. 8.5% of male KTS patients may be associated with scrotal vascular malformations, and 9.5% of female KTS patients may have vulva and vaginal vascular malformations.^[6]^ And bladder hemangioma is very common, mostly located in the anterior wall and the top of the bladder. Under cystoscope, dark red, pedicled or unpredicted, lobulated abnormal blood vessels can be seen. Trigone of the bladder is rarely involved.^[13]^ Abnormal development of lymphatic vessels can be manifested as lymphangiomatous changes or recurrent erysipelas. The main principle of bladder hematuria is that the abnormally thickened permanent ischial vein in KTS patients causes pelvic vein overload, hindering internal iliac vein return, and eventually thickening and rupturing the bladder vein.

Most of the hematuria of KTS can be relieved by conservative treatment.^[14]^ The onset age of hematuria in this patient is relatively late, and only small varicose veins in the mucosa can be seen under cystoscope and flexible ureteroscope. Small hemangioma protrudes from the bladder mucosa, and no large hemangioma and thick veins can be seen, so the degree of hematuria is mild. The final symptoms also improved significantly, follow-up so far has not recurred. Husmann DA noted that recalcitrant gross hematuria from urethral, bladder and upper tract sources could be managed safely by endoscopic and angiographic methods, saving 60% of the patients from more aggressive and moribund open surgical intervention.^[15]^ However, in the case of poor conservative treatment, such patients often have thick drainage veins and extensive vascular malformation in the pelvic cavity. Although hemostasis is not completely possible, internal iliac artery embolization can temporarily reduce bladder bleeding, save lives, and create conditions for subsequent cystoscopy and laser therapy.^[4]^ Currently, it is believed that endoscopic laser can produce deep thermal effect and good tissue coagulation effect, which has become the preferred method for the treatment of hemangiomatoid lesions in the bladder.^[7]^ Nd : YAG laser is the most suitable type. Of course, 810 nm high-power semiconductor laser should be able to treat such diseases theoretically. Partial cystectomy can be performed for large local lymphohemangiomatous hemorrhage, and subtotal cystectomy should be considered for extensive intravesical hemorrhage. Endoscopic focal resection is not recommended at present, because it is difficult to estimate the scope and depth of abnormal blood vessels under the microscope, which may induce uncontrollable massive bleeding.^[14]^ As for limitations,in this case, no arteriography was performed to distinguish patients from Parkes-Weber syndrome, and interventional therapy was not used to evaluate the treatment of malformed veins and hemangioma. On the other hand, we may be able to delay or control the progression of the disease as much as possible through gene therapy.

## 4. Conclusion

If the clinical experience of unexplained hematuria, or the onset of hematuria is younger, especially in childhood, and if the characteristics of lower extremity varicose veins, skin wine pigment spots, bone and soft tissue hypertrophy, we need to consider the possibility of urinary system involvement of KTS. Cystoscopes and ureteroscopes often present with hemangioma of bladder and diffuse hemorrhagic sites of calyces and pelvis. Most of them can be improved by conservative treatment. Cystoscopic laser treatment is the first choice for bladder bleeding. When bleeding is threatening, partial and subtotal cystectomy and nephrectomy should also be considered.

## Author contributions

**Data curation:** Feng Lin, Kewei Yang, Jiadong Xu, Lixia Yang, Jinrong Huang.

**Investigation:** Feng Lin, Jiadong Xu, Gang Wang, Lixia Yang.

**Supervision:** Kewei Yang.

**Writing – original draft:** Feng Lin, Kewei Yang, Jiadong Xu, Gang Wang, Dan Li.

**Writing – review & editing:** Feng Lin, Kewei Yang, Dan Li.
